# 695. Incidence and Risk Factors for *Clostridioides difficile* Infections in Non-COVID and COVID-19 Patients

**DOI:** 10.1093/ofid/ofad500.757

**Published:** 2023-11-27

**Authors:** Therese Mathew, Rhema Maria Punnoose

**Affiliations:** St. Joseph's College of Pharmacy, Kollam, Kerala, India; St. Joseph's College of Pharmacy, Kollam, Kerala, India

## Abstract

**Background:**

Infections with *Clostridioides difficile* (CDIs) are a leading cause of healthcare-associated infections (HAIs) and impact public health globally. The aim of this study was to assess the incidence and the risk factors for healthcare-associated *Clostridioides difficile* infection (HA-CDI) in patients with COVID-19 and without this infection.

**Methods:**

A single-center, prospective observational study was conducted at a tertiary care hospital in India, from January 2021 to December 2022. The entire hospital was a COVID-dedicated hospital for 12 months during the study period. The incidence density rates and risk factors for HA-CDI in patients with and without COVID-19 are presented.

**Results:**

The incidence rates of HA-CDIs were three times higher in patients with COVID-19. The HA-CDI–COVID-patients were younger (69.9 ± 12.6 vs. 72.5 ± 11.6; *p* = 0.017), admitted from another hospital (20.5% vs. 2.9; *p* < 0.001), had antimicrobial therapy before CDI (99.1% vs. 91.3%, *p* < 0.001), received two or more antibiotics (*p* = 0.030) during a longer period (p = 0.035), received proton pump inhibitors (95.9% vs. 50.0%, *p* < 0.001) during a longer period (*p* = 0.012) and steroids (32.8% vs. 20.4%, *p* < 0.001). During the last month before their current hospitalization, a higher percentage of patients without COVID-19 disease were hospitalized in our hospital (*p* < 0.001). Independent predictors for HA-CDIs in patients with COVID-19 were admission from another hospital (*p* = 0.003), the length of antibiotic administration (0.020), and the use of steroids in therapy (*p* < 0.001). The HA-CDI predictors in the non-COVID patients were older age (*p* = 0.017), advanced-stage renal failure (*p* = 0.005), chemotherapy (*p* = 0.003), and a low albumin level (0.005).

**Conclusion:**

Higher incidence rates of HAI-CDIs in COVID-19 patients did not occur due to reduced infection control precautions and hygiene measures but due to antibiotic therapy and therapy with other drugs used during the pandemic.

**Disclosures:**

**All Authors**: No reported disclosures

